# T cell receptor recognition of CD1b presenting a mycobacterial glycolipid

**DOI:** 10.1038/ncomms13257

**Published:** 2016-10-27

**Authors:** Stephanie Gras, Ildiko Van Rhijn, Adam Shahine, Tan-Yun Cheng, Mugdha Bhati, Li Lynn Tan, Hanim Halim, Kathryn D. Tuttle, Laurent Gapin, Jérôme Le Nours, D. Branch Moody, Jamie Rossjohn

**Affiliations:** 1 Infection and Immunity Program, Biomedicine Discovery Institute, Monash University, Clayton, Victoria, Australia; 2 Department of Biochemistry and Molecular Biology, Monash University, Clayton, Victoria, Australia; 3 Australian Research Council Centre of Excellence in Advanced Molecular Imaging, Monash University, Clayton, Victoria, Australia; 4 Division of Rheumatology, Immunology and Allergy, Brigham and Women's Hospital, Harvard Medical School, Boston, Massachusetts, USA; 5 Department of Infectious Diseases and Immunology, Faculty of Veterinary Medicine, Utrecht University, Utrecht, The Netherlands; 6 Department of Immunology and Microbiology, University of Colorado Anschutz Medical Campus and National Jewish Health, Aurora, Colorado, USA; 7 Institute of Infection and Immunity, Cardiff University School of Medicine, Cardiff, UK

## Abstract

CD1 proteins present microbial lipids to T cells. Germline-encoded mycolyl lipid-reactive (GEM) T cells with conserved αβ T cell receptors (TCRs) recognize CD1b presenting mycobacterial mycolates. As the molecular basis underpinning TCR recognition of CD1b remains unknown, here we determine the structure of a GEM TCR bound to CD1b presenting glucose-6-*O*-monomycolate (GMM). The GEM TCR docks centrally above CD1b, whereby the conserved TCR α-chain extensively contacts CD1b and GMM. Through mutagenesis and study of T cells from tuberculosis patients, we identify a consensus CD1b footprint of TCRs present among GEM T cells. Using both the TCR α- and β-chains as tweezers to surround and grip the glucose moiety of GMM, GEM TCRs create a highly specific mechanism for recognizing this mycobacterial glycolipid.

## Abstract (web summary)

Germline-encoded mycolyl lipid-reactive (GEM) T cells recognize CD1b proteins presenting mycobacterial mycolates via their T-cell receptors (TCRs). Here, the authors present the structure of this interaction and provide a molecular basis for the co-recognition of CD1b and a mycobacterial glycolipid.

## Graphical abstract

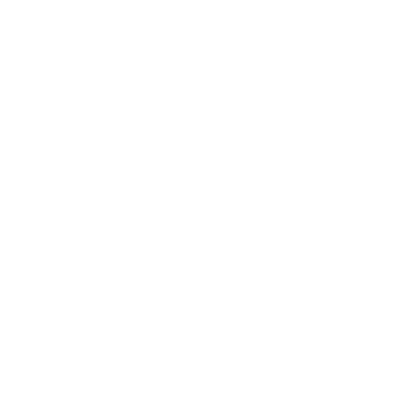

αβ T cells use their clonotypic αβ T cell antigen receptors (TCR) to sense microbial-derived peptides that are presented by molecules encoded by the polymorphic major histocompatibility complex (MHC)^1^. More recently, studies show that TCRs can recognize foreign non-peptide antigens, including bacterial vitamin B metabolites bound to MR1 and microbial lipid-based antigens bound to CD1 (refs 2, 3, 4). These studies broadly expand the biochemical range of natural antigens that trigger αβ T cell responses.

A second new and general insight into αβ T cell function is that the monomorphic CD1 and MR1 antigen-presenting molecules can activate conserved populations of responding T cells, which are not restricted to the genetic background of the donor. Such ‘donor-unrestricted T cells' can show characteristic TCR gene usage patterns that are more broadly conserved across the human species than even the most public MHC-restricted TCRs^3^,^5^,^6^,^7^. The two most widely studied examples are type I Natural Killer T cell (NKT) TCRs, which typically express TCR α-chains encoded by the TRAV10-TRAJ18 gene segments and recognize CD1d (ref. 8), and mucosal-associated invariant T cells (MAIT) that are typically encoded by TRAV1-2 joined to TRAJ33 and recognize MR1 (refs 9, 10).

In addition to CD1d, which is designated as a group 2 CD1 protein, the human *CD1* locus encodes three group 1 antigen-presenting molecules, CD1a, CD1b and CD1c. Each of these proteins possesses distinctly shaped antigen-binding clefts and show differing expression on B cells, myeloid dendritic cells and Langerhans cells, which increasingly point to separate immunological functions^11^,^12^,^13^,^14^,^15^. CD1b differs from the other human CD1 proteins in that it binds both to adaptor protein 2 (AP-2) and AP-3 complexes, which promote trafficking to lysosomes, where antigen loading is more strongly controlled by acid pH (ref. 16). Also, CD1b possesses a particularly large and deep antigen-binding cleft that contains two pockets (C′, T′) not found in other CD1 proteins. The four antigen-binding pockets (A′, F′, C′ and T′) of CD1b bind the hydrocarbon chains of amphipathic antigens (Ag), allowing the hydrophilic head groups to protrude from the F′-pocket through the F′-portal^4^. The outer surface of CD1b near the F′-portal is the presumed surface for TCR contact, but the mode of TCR binding to CD1b has not been directly observed.

The development of group 1 CD1 tetramers now increases our understanding of human lipid-reactive T cell populations^17^,^18^,^19^. For example, CD1b tetramers carrying a mycobacterial glycolipid, glucose-6-*O*-monomycolate (GMM), demonstrated the existence of polyclonal T cells recognizing GMM lipids and among tuberculosis (TB) patients^20^,^21^. CD1 proteins are non-polymorphic and the responding T cells show two defined TCR conservation patterns. Namely, germline-encoded mycolyl lipid-reactive (GEM) T cells express nearly identical TCR α-chains encoded by TRAV1-2 and TRAJ9, and TCR β-chains that are biased toward usage of TRBV6-2 or TRBV30 (ref. 20). This TCR α-chain was also identified in one T cell clone (clone 18) that recognizes free mycolic acid, a deglycosylated form of GMM. In addition, LDN5-like T cells are a distinct T cell population that expresses TRBV4-1^+^ TCRs, which bind CD1b–GMM complexes with lower affinity than the GEM TCRs^22^. Thus, TCR-defined T cell types exist in the human CD1b-reactive repertoire. Here we describe the structure of a GEM TCR bound to the CD1b–GMM complex, thereby representing the first description of a TCR–CD1b–Ag ternary complex and sheds light on the general nature lipid-reactive TCRs that are broadly conserved in humans^21^. These data provide specific structural explanations for the TCR variable (V) and joining (J) genes that define GEM T cells, identify two distinct modes of typical and atypical antigen recognition, as well as conceptual insight into the biased TCR selection of GEM T cells towards a glycolipid antigen by a pathogen of worldwide importance.

## Results

### Overview of the GEM TCR–CD1b ternary complex

GEM TCRs from clones 1, 42, 21 bind CD1b–GMM with relatively high affinity (K_D_≈1 μM)^20^. We refolded the TCR from clone 42 (GEM42) that was encoded by three gene segments typical of GEM TCRs: TRAV1-2, TRAJ9 and TRBV6-2 (Supplementary Table 1). Next we generated a panel of CD1b mutants, of which one (Ile160Ala) was expressed at a particularly high yield. Like wild type CD1b, CD1b-Ile160Ala readily loaded a natural GMM with an average chain length of C32 (C32 GMM) and bound the GEM42 TCR with a comparable but slightly higher affinity that wild type CD1b. We subsequently determined the structure of the ternary complex to 3.2 Å resolution (Table 1; Supplementary Fig. 1).

**Figure 1:**
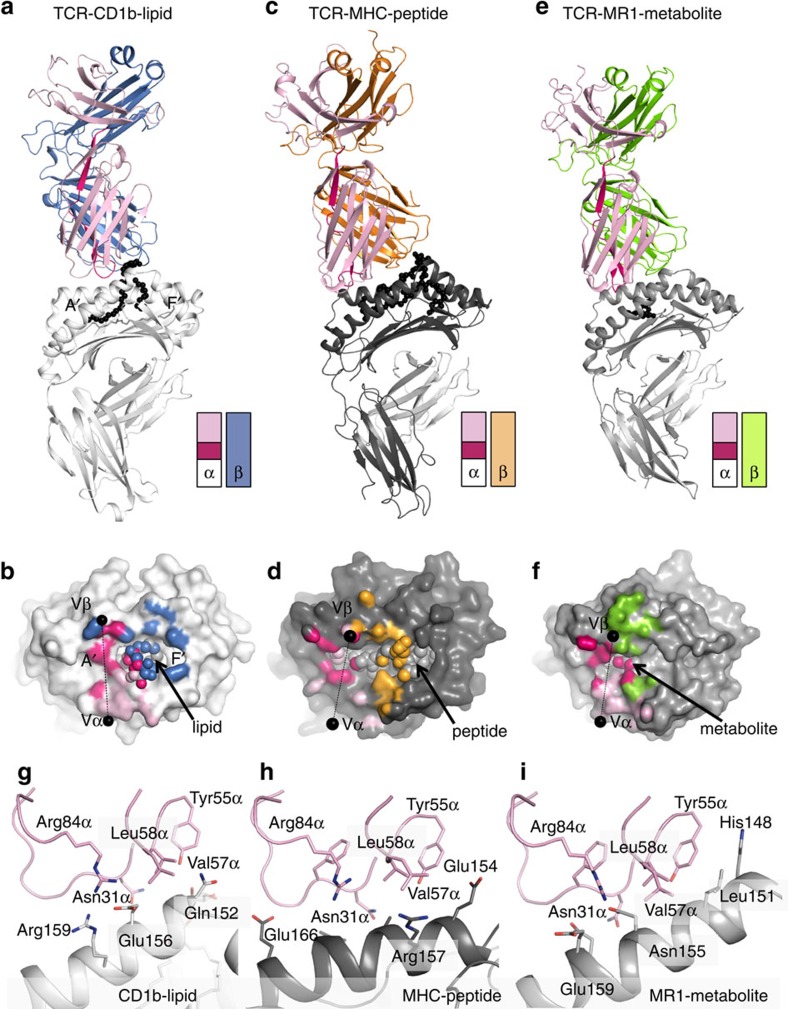
TRAV1-2 TCRs recognize CD1b, MHC-I and MR1. Overview of the TRAV1-2 TCRs in complex with CD1b (coloured white; **a**,**b**,**g**), MHC-I (coloured dark grey; **c**,**d**,**h**) and MR1 (coloured light grey; **e**,**f**,**i**) molecules. The top panels show the overview of each complex represented in cartoon format with the antigen in black spheres. The TRAV1-2 gene segment is coloured in light pink, the TRAJ gene segment in vibrant pink, and the β-chain in blue, orange and green for GEM42 TCR (**a**,**b**), ELS4 TCR (**c**,**d**) and MAIT TCR (**e**,**f**), respectively. A schematic of each TCR gene segment is represented as two rectangles for the α and β-chains, with TRAV1-2, TRAJ and the β-chains coloured as per the top panels. The middle panels show the footprint of each TCR on the surface of the CD1b-lipid (**b**), MHC-peptide (**d**) and MR1-metabolite (**f**). The black spheres on the middle panels represent the centre of mass of the Vα and Vβ domains, while the light grey spheres represent the antigen bound in each molecule. The atomic footprint is coloured according to the TCR segment making contact. The bottom panels show the TRAV1-2 gene segment (light pink) contact with (**g**) CD1b-lipid (white), (**h**) MHC-peptide (dark grey) and (**i**) MR1 (light grey).

**Figure 2:**
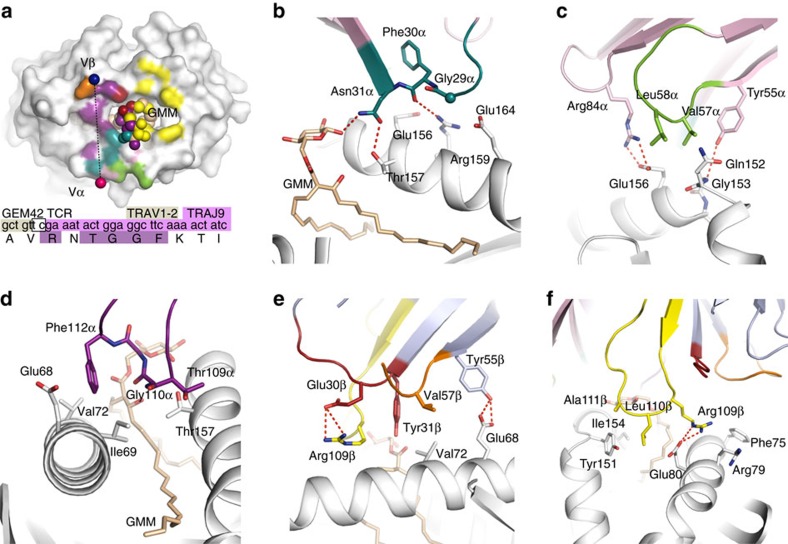
GEM TCR footprint on CD1b–GMM. (**a**) Footprint of the GEM42 TCR on the surface of CD1b (white) and GMM (pale orange spheres) is represented according to the atoms contacted and coloured as per the TCR segment making contact. Framework residue from the α-chain in pale pink, the CDR loops coloured in teal (CDR1α), green (CDR2α) and purple (CDR3α) for the α-chain and red (CDR1β), orange (CDR2β) and yellow (CDR3β) for the β-chain, respectively. Pink and blue spheres represent the centre of mass of the GEM42 TCR α and β-chains, respectively. The insert below the footprint represents the characteristic CDR3α loop sequence of the GEM TCR. GEM42 TCR interactions with the CD1b (panels **b**–**f**), with the CD1b in white, GMM in pale orange and the GEM 42 TCR coloured as per panel (**a**). The panels represent residues from the (**b**) the CDR1α (teal); (**c**) CDR2α (green) and framework residue from α-chain (pale pink); (**d**) CDR3α (purple); (**e**) CDR1/2β (red and orange) and framework β-chain (pale blue); (**f**) CDR3β (yellow) interacting with the CD1b molecule (white). Hydrogen bonds are shown as red dashed lines, and the sphere represents the Cα atom of the glycine 29α residue.

The GEM42 TCR docked over the α1 and α2-helices of CD1b with a centrally located footprint (Fig. 1). A comparison of the first footprints of TCRs on CD1a and CD1b show an extreme contrast. Namely, the CD1a autoreactive TCR (BK6) binds at a site distant from the F′-portal of CD1a and does not contact the bound lipid ligands^23^ (Supplementary Fig. 2). Instead, the GEM42 TCR is positioned near the centre of the CD1b platform and directly covers the F′-portal, thereby fully surrounding and extensively contacting the protruding glucose headgroup (Fig. 1b). Specifically, the GEM TCR bound in an orthogonal orientation with respect to the long axis of the CD1b antigen-binding cleft, whereupon the TCR α-chain and β-chains sat over the α2-helix (residues 151–160) and α1-helix (residues 68–80) of CD1b, respectively (Fig. 1b; Supplementary Table 2). The buried surface area (BSA) on complexation by the GEM TCR was ∼1,600 Å^2^, of which the TCR α- and β-chains contributed 52% and 48%, respectively. At this interface, the CDR3α loop and CDR3β loop contributed the most to the interaction with CD1b–GMM, with 27% and 35% of BSA, respectively (Figs 1b and 2a). Thus, the GEM TCR formed an extensive interaction network with CD1b–GMM complex, thereby providing immediate molecular insight into the basis for the previously observed patterns of TCR α- and β-chain conservation in polyclonal GEM T cells from latent TB patients^20^.

### Role of GEM TCR α-chain in CD1b recognition

Critical questions we aimed to address were whether and how GEM-defining TRAV and TRAJ regions control specificity for CD1b and GMM. The TRAV1-2 gene element encodes the CDR1α and CDR2α germline-encoded loops, which played an important role (10% BSA each) in contacting CD1b–GMM. In contrast, the corresponding CDR1β and CDR2β loops from the TCR β-chain played a lesser role (BSA of 5% and 4%, respectively) (Fig. 2a; Supplementary Table 2). These differential contributions from the TRAV and TRBV regions of the GEM TCR reflected the different extent of the TCR α- and β-chain bias in GEM T cells.

Considering the particular roles of GEM TCR-defining residues in CD1b–GMM recognition, the positioning of Gly29α and Phe30α enabled the CDR1α loop to lay flat and proximal to the α2-helix of CD1b, with the main chain backbone of the CDR1α loop mediating van der Waals and polar contacts with a cluster of CD1b residues including Glu156, Arg159 and Glu164 (Supplementary Table 2). These interactions were complemented by the Asn31α side chain forming a hydrogen bond with Thr157 from CD1b (Fig. 2b). The TRAV1-2-encoded CDR2α loop mediated hydrophobic contacts with CD1b, whereby Val57α and Leu58α wedged between the aliphatic side chains of Gln152 and Glu156 of CD1b (Fig. 2c). These hydrophobic contacts were flanked by two closely associated framework residues, Tyr55α and Arg84α from the TRAV1-2 chain, which interacted with CD1b. Here, Tyr55α is within hydrogen bonding distance to the main chain of CD1b (Gly153), and Arg84α extended towards CD1b and formed a salt bridge with Glu156 (Fig. 2c).

The TRAJ9-encoded CDR3α loop played a key role at the GEM TCR–CD1b–GMM interface, abutting the GMM antigen, and residing between the helical jaws of the CD1b antigen-binding cleft, forming mostly hydrophobic contacts with residues from both the α1- and α2-helices of CD1b. Here, Thr109α and Gly110α packed against Thr157 from the α2-helix of CD1b, while Phe112α plugged into a hydrophobic crevice formed by Ile69, Val72 and the aliphatic moiety of Glu68 (Fig. 2d). In comparison to the other CD1 isoforms, the invariant TCR α-chain of the GEM TCR made a series of contacts that were either unique to CD1b, or absent in some of the other CD1 family members (Supplementary Fig. 3).

### Role of the TCR β-chain in CD1b recognition

The role of the CDR1β loop was restricted to Tyr31β contacting Val72 on CD1b, the latter of which was also contacted by Val57β from the CDR2β loop (Fig. 2e). This CDR2β loop contact was supported by an adjacent framework residue Tyr55β, which hydrogen-bonded to Glu68 of CD1b (Fig. 2e). Thus the germline-encoded regions of the TRBV6-2 gene played a limited role in contacting CD1b (Fig. 2a and Supplementary Table 2), consistent with its present, but weak contribution to GEM TCR motifs observed in polyclonal T cells. Nevertheless, Glu30β from the CDR1β loop, while not contacting CD1b, played a key role in maintaining the conformation of Arg109β from the CDR3β loop, which formed many contacts with GMM and CD1b (Supplementary Table 2). Here, Arg109β extended into a niche formed by Phe75, Arg79 and Glu80, forming a salt bridge with Glu80 from CD1b (Fig. 2f). Glu80 abutted Leu110β, which, together with Ala111β also stacked against Tyr151 from CD1b (Fig. 2f). Collectively, our observations suggest a potential role of the CDR3β loop in fine-tuning the interactions with the F′-pocket of CD1b, thereby refining GEM TCR recognition of CD1b–GMM (ref. 20).

### A two-chain tweezers mechanism grips GMM

**Figure 3:**
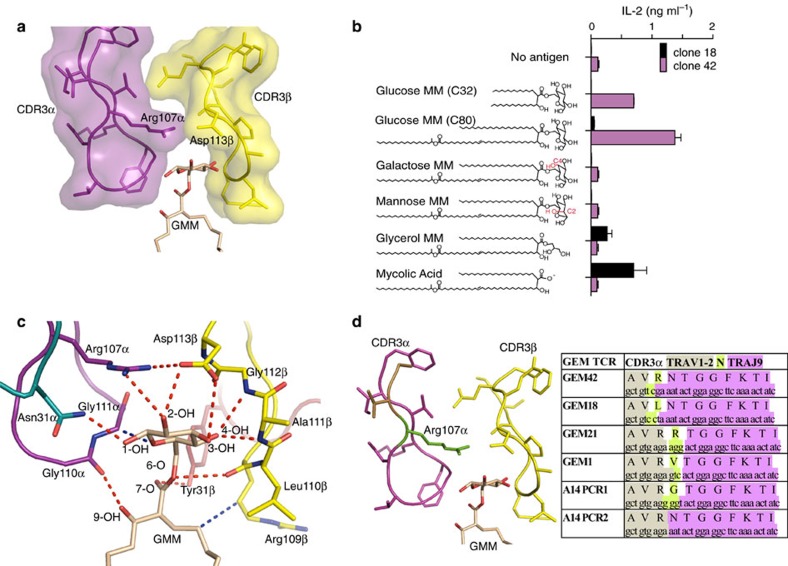
Molecular tweezers grip the glucose headgroup of GMM. (**a**) Surface representation of the CDR3α (purple) and CDR3β (yellow) loops acting as ‘tweezers' grasping the GMM glucose head group (pale orange stick). (**b**) Schematic of the GMM analogues tested against the GEM T cells. Red shows the different hydroxyl orientation of Galactose monomycolate (MM) and Mannose MM. Each GMM analogue was used to stimulate IL-2 production upon presentation to GEM clone18 (black bar) and GEM clone 42 (pink bar), error bar representing triplicate wells. The experiments was performed twice with similar results. (**c**) Interaction network between the GMM (pale orange stick) and the GEM42 TCR (coloured as per Fig. 2a). The red dashed lines represent hydrogen bonds, while the blue dashed line represent hydrophobic interactions. (**d**) CDR3α loop sequences of the GEM TCR, element from the TRAV1-2 gene are highlighted in sand colour, N region in green and TRAJ9 gene in pink, while the CDR3β loop is coloured in yellow as per panel **c**.

The mechanism of glycolipid recognition by the GEM TCR is visually striking and can be compared to tweezers, whose jaws are formed by the CDR3α and CDR3β loops, grasping at the protruding glucosyl moiety of GMM (Fig. 3a). This headgroup grasping mechanism is highly distinct from a recently solved CD1a–lipid–TCR complex^23^, in which the TCR does not directly contact bound lipid. Further, this two-chain mechanism in which glucose is sandwiched between TCR α- and β-, is distinct from type I and type II NKT TCR antigen recognition, in which only one TCR chain contacts antigen^24^,^25^,^26^,^27^. This tweezers mechanism prompted functional studies of the GEM TCR's fine specificity for GMM.

Whereas most GEM T cells recognize GMM, the extent of fine specificity for antigen structure remains unknown. One ‘atypical' GEM TCR expressed by clone 18 (GEM18 TCR) recognized free mycolic acid (MA). This antigen lacks the glucosyl moiety, pointing toward possible promiscuity^20^. On the other hand, CD1b-reactive clones known as LDN5-like T cells, do show specificity for the glucosyl group of GMM^22^,^28^. We measured GEM clone 42 and clone 18 responses to molecules with altered head groups, including MA, which lacks glucose, as well as mannose-6-*O*-monomycolate (ManMM) and galactose-6-*O*-monomycolate (GalMM), which differ from GMM only in the orientation of one hydroxyl group in the hexosyl ring at position C2 or C4, respectively. In all cases alteration of the headgroup abolished the GEM clone 42 T cell response, demonstrating precise specificity for the glucosyl moiety (Fig. 3b).

This fine specificity for GMM arose from the extensive contacts made by the GEM42 TCR. Here, the double glycine motif, Gly110α-Gly111α, enabled the CDR3α loop of the GEM42 TCR to pack against the glucose headgroup, while the main chain carbonyl of Gly110α also formed a hydrogen bond with the mycolyl β-hydroxyl group of GMM (Fig. 3c). In addition, Arg107α rested above GMM, where it hydrogen-bonded with the 2-OH moiety of the glucose headgroup. The axial position of 2-OH in ManMM likely accounted for the lack of recognition by the GEM42 TCR (Fig. 3b). Moreover, Arg107α ‘collaborated' with Asp113β from the CDR3β loop, by forming a salt bridge with it, which held Asp113β in place so that it formed hydrogen bonds with the 2-OH and 3-OH groups of glucose (Fig. 3b). Thus, Arg107α functioned like a ‘capstone' as its positioning allowed two key interdomain interactions: stabilization of the TCR α and β-chains, as well as binding with glucose. The lack of response to GalMM and other related analogues correlated with the finding that glucose moiety formed hydrogen bonding contacts with the main chain of the CDR3β loop (Supplementary Table 2). While there is space to accommodate a galactose moiety between the molecular tweezers, the altered stereochemistry of the C4 hydroxyl would result in loss of interactions with the TCR β-chain at positions Ala111β and Gly112β and unfavourable contacts with CD1b.

GMM was also contacted by other germline-encoded elements of the GEM42 TCR. Namely, Asn31α from the CDR1α loop formed a hydrogen bond with the C1 hydroxyl moiety of GMM, and Tyr31β from the CDR1β loop formed a hydrogen bond with the carbonyl oxygen of the glucosyl ester linkage to mycolic acid (Fig. 3c). As such, every polar moiety of the GMM antigen was involved in an interaction with the GEM42 TCR (Fig. 3c). Indeed, of the 230 Å^2^ solvent exposed surface area of GMM available for TCR contact, 93% was buried upon ligation with the TCR. Accordingly, the tweezers-like grip of the GMM antigen provided the molecular basis for the high affinity and strict specificity of this GEM42 TCR for this mycobacterial glycolipid.

### Roles of germline and N-region encoded residues in GEM TCRs

GEM T cells were so named based on the use of germline-encoded sequences and the paucity of N-region additions at the TRAV–TRAJ junction. Comparison of the CDR3α sequences from GEM T cells suggests a candidate mechanism by which N-region additions, when present, do not interfere with CD1b–GMM binding. In half of the known GEM TCR α-chains, position 108 is encoded by N nucleotides. This residue is variable, and can be Asn, Arg, Val or Gly (Fig. 3d). This relative insensitivity of GEM TCRs to changes encoded by N-region additions at position 108α agrees with the structural observation that this residue is rotated outward on the CDR3 loop so that it makes no interactions with TCR β-chain, CD1b or GMM.

The Arg107α is conserved among all GMM-specific GEM TCRs. Arg107 is encoded by TRAV1-2 in GEM clones 21 and 1, as well as two TCRs from donor A14, known as A14PCR1 and A14PCR2 (ref. 20). In one of the GEM TCR α-chains, A14PCR2, the consensus GEM CDR3α is formed without any N nucleotides, and thus entirely encoded by the genome. However, in the GEM42 TCR, TRAV1-2 was trimmed so that it lost sequences that would have encoded Arg107α, but the N nucleotides encoded Arg107α (Fig. 3d). Combining these observations with the structural evidence that Arg107α serves a capstone function (Fig. 3a), we propose a capstone switch mechanism whereby TRAV1-2 usually encodes Arg107α. In the case that nucleotides encoding this residue are chewed back, the relatively rare event of random N-nucleotide recovery of arginine can nevertheless be selected based on the crucial structural role of the arginine side chain.

### Typical and atypical GEM TCRs

The GEM18 TCR can be considered atypical, because it recognizes unglucosylated mycolic acid whereas the other typical clones recognize GMM. Clone 18 differs from all five typical GEM TCRs in that it lacks arginine at position 107α, and possesses a Leu107α instead, thereby explaining the lack of specificity for glucose (Fig. 3d). Clone 18 also lacks the key Asp113β, which is found in three typical GEM TCRs and contributes TCR α and β pairing^20^ (Supplementary Table 1). Conversely, clone 18 does possess two residues, Gly110α and Phe112α, which interact with the A′-roof of CD1b and aspects of the mycolyl moiety that are shared between GMM and MA. Thus, the GEM42 TCR provides a framework for identifying the particular mechanisms of atypical TCR mediates recognition of mycolic acid.

### Remoulding of CD1b–GMM on GEM TCR ligation

**Figure 4:**
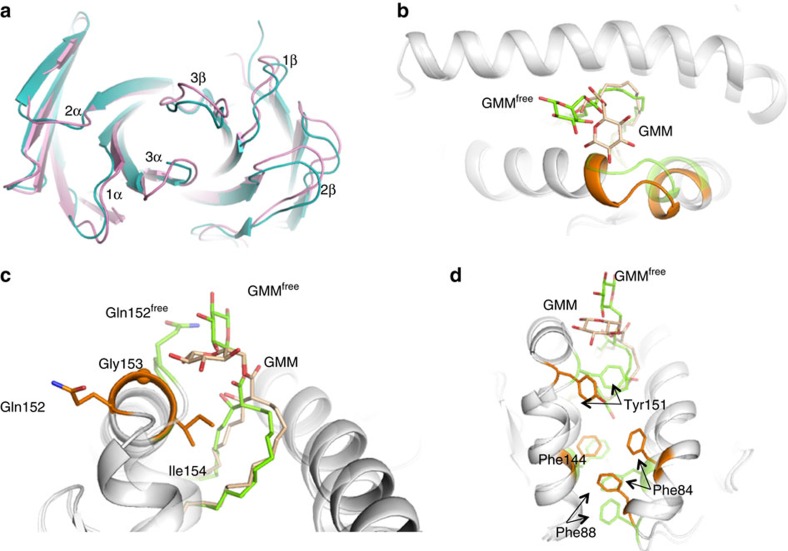
CD1b–GMM plasticity upon GEM TCR engagement. (**a**) Superposition of the GEM42 TCR free (blue) and bound to CD1b–GMM (pink) structures. (**b**–**d**) Superposition of the binary CD1b–GMM (green) and bound to GEM42 TCR (orange). GMM is represented as stick, and CD1b in cartoon format, while the sphere represents the Cα atom of the glycine 153 residue. The orange and green colours highlight the segment of the CD1b molecule that change conformation upon binding of the GEM42 TCR, namely residues 147–154 of the α2-helix (**b**,**c**) and the aromatic residues of the F′-pocket Tyr151, Phe144, Phe88 and Phe84 (**d**).

To establish the extent of conformational plasticity occurring upon TCR engagement of CD1b–GMM, we compared the GEM42 TCR–CD1b–GMM ternary complex to the binary CD1b–GMM (C32) structure (Table 1, Supplementary Fig. 4), and the previously solved structure of the non-liganded GEM42 TCR (ref. 20). With the exception of the CDR3β loop, the CDR loops of the GEM TCR did not move appreciably after CD1b–GMM binding. The CDR3β loop moved maximally by∼4 Å to avoid steric clashes and make favourable contacts with CD1b–GMM (Fig. 4a). Thus, the invariant TCR α-chain of the GEM TCR was a fixed recognition determinant of CD1b–GMM. Our binary structure of CD1b-C32 GMM was similar to one previously determined with C54 GMM (C51-C57) bound to CD1b (ref. 29) (Supplementary Fig. 5). The glucosyl moiety extends substantially out of the CD1b cleft and can exhibit mobility (Supplementary Fig. 4). TCR engagement caused two major conformational changes. Namely, the glucose headgroup was flattened by the CDR3α loop, being displaced by ∼5 Å towards the α2-helix of CD1b (Fig. 4b,c). This bulldozing of the headgroup was essential to enable favourable contacts between glucose and the GEM TCR, and also enabled TCR contacts with the CD1b molecule itself (Fig. 4c). To accommodate the altered position of GMM, the hinge region of the CD1b α2-helix was remoulded (Fig. 4c). Repositioning of Gln152 caused a ripple of conformational change, including the displacement of Tyr151, which caused re-orientation of Phe84 and Phe88 as well as shifting of Phe144 (Fig. 4d). Collectively these changes altered a segment of the α2-helix of CD1b itself (residues 146–150), undergoing a rigid body shift of 1.5–2.0 Å (Fig. 4b). Thus a relatively rigid GEM TCR forced a series of large conformational changes in GMM and CD1b to enable high affinity engagement.

### TRAV1-2 recognition of MHC-I, MR1 and CD1b

TRAV1-2 gene usage is a defining feature of polylconal TCRs present on both MAIT cells and GEM T cells, and it is present in some MHC-reactive TCRs^20^,^30^,^31^. In all three corresponding TCR ternary complexes^30^,^32^ (Fig. 1; Supplementary Table 3), a large part of the footprint was made by the germline-encoded TCR α-chain, highlighting the basis for the TRAV1-2 usage. The axis of orientation (80–90°) and the position of the footprint of TRAV1-2-encoded regions were similarly placed atop the MHC-I, MR1 and CD1 platforms, a remarkable finding given the lack of sequence homology in the three footprint regions.

Next we carried out a comparative analysis of the surface interactions to identify shared or distinct mechanisms of binding, emphasizing CD1b and MR1 as the two non-polymorphic proteins that generate species-wide patterns of TCR response (Fig. 1; Supplementary Table 3). One identifiable shared aspect of recognition was notable in the CDR1α loop, where all three TCRs used Asn31α to engage the respective antigen-presenting molecules (Fig. 1; Supplementary Table 3). Also, the CDR2α loop docked a similar portion of the CD1b, MHC and MR1 molecules. TRAV1-2 encodes a hydrophobic motif of the CDR2α loop (Val57-Leu58), which forms a ‘clamp' that docked onto the α2-helix of CD1b, MR1 and MHC. These interactions are further helped by the framework residue Tyr55α, which packs its aromatic ring with the ‘hydrophobic clamp' and directly contacts the CD1b, MHC and MR1 molecules. Upstream of this hydrophobic patch, Arg84α points towards the α2-helix and is forming either a salt bridge with CD1b (Glu156) or hydrogen bond with MR1 (Asn155) and MHC (Gly162). Overall the TRAV1-2 exhibits a docking mimicry with three evolutionarily conserved antigen presenting molecules.

### TCR energetic footprint on CD1b

**Figure 5:**
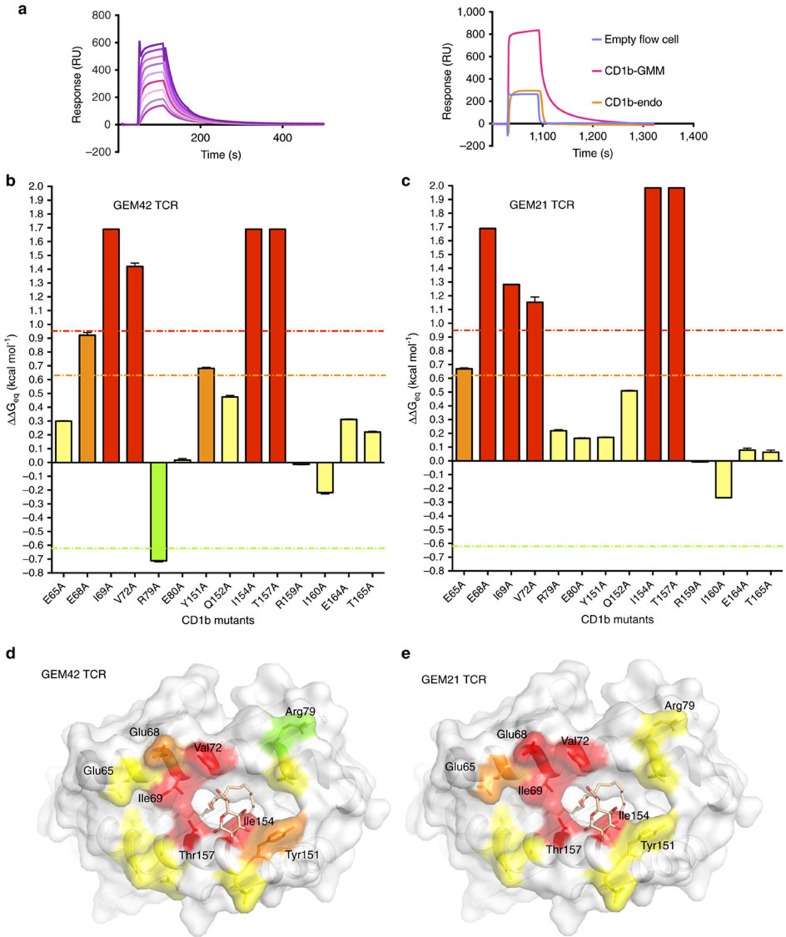
Energetic hotspot of GEM TCR–CD1b–GMM recognition. (**a**) Surface plasmon resonance (SPR) sensogram of CD1b–GMM interacting with a range of GEM42 TCR concentrations (top graph represented by the purple and pink curves), as well as the sensogram of GEM42 TCR passed over an empty flow cell used as control (blue curve), CD1b loaded with endogenous lipid (orange curve) or GMM (pink curve). (**b**,**c**) SPR on the CD1b mutants loaded with GMM with the GEM42 TCR (**b**) and GEM21 TCR (**c**). The experiments were performed in duplicate (*n*≥2), with the error bar representing the standard deviation. The *y*-axis represents the ΔΔGeq (kcal mol^−1^), the error bar represents standard deviation, the orange line indicates a 3-fold increase compared the wild type protein; the red line indicates a 5-fold increase; and the green line represents a 3-fold decrease. The *x*-axis indicates the position of CD1b mutation, and the bars are coloured according to the impact of each mutation on the affinity of the TCRs, ranging from 3-fold improvement (green), no impact (yellow), 3-fold decrease (orange) and more than 5-fold decrease (red). (**d**,**e**) Surface representation of CD1b (white) and GMM (pale orange stick) with the mutated residues coloured as per their impact on the GEM42 (**d**) and GEM21 TCR binding (**e**), coloured as per panels (**b**,**c**).

To determine the role of residues near the entrance to the CD1b cleft, we made six single-site alanine mutants of the α1-helix and eight mutants of the α2-helix. Each CD1b mutant was loaded with GMM, and assessed by surface plasmon resonance (SPR) for binding the GEM42 TCR (TRBV6-2^+^) and the GEM21 TCR (TRBV30^+^). These two GEM TCRs use the two most common TCR β-chain variable genes seen among polyclonal GEM T cells^20^ (Supplementary Table 1). The GEM42 and GEM21 TCRs possess an affinity (K_D_) of 1.15 and 0.70 μM for CD1b–GMM (Supplementary Table 4), respectively, and lacked detectable affinity towards the untreated CD1b (Fig. 5a). Compared to wild type CD1b, the CD1b-Ile160Ala mutant slightly improved the affinity of both GEM42 and GEM21 TCRs (Supplementary Table 4; Supplementary Fig. 6a). An alanine at position 160 might allow the GEM TCR to approach closer to the CD1b–GMM complex, thereby favouring the interaction (Supplementary Fig. 6b).

For the GEM42 TCR, one mutant improved (3–5 fold), seven had no impact (<3-fold change), two had moderate impact (3–5 fold reduction), and four (Ile69Ala, Val72Ala, Ile154Ala and Thr157Ala) were critical (>5-fold reduction) to affinity (Fig. 5b). All four critical residues form contacts with the CDR1α and CDR3α loops of the invariant TCR α-chain or the CDR3β loop, and two of the critical residues, Ile154 and Thr157, interacted with the GMM headgroup (Fig. 2f; Supplementary Fig. 7). For the GEM21 TCR, these same four CD1b mutants were critical (Fig. 5c, Supplementary Table 4), indicating a common energetic ‘hot spot' for these two GEM TCRs.

Notably, however, CD1b residues located near the F′-pocket had different effects on binding the two TCRs (Fig. 5d,e). Namely, the Arg79Ala and Tyr151Ala mutants increased and moderately decreased the affinity of the interaction towards the GEM42 TCR (Fig. 5b,d), respectively, but these mutants had no impact on the affinity of the interaction for the GEM21 TCR (Fig. 5c,e). These residues mapped to the site of the interaction with the hypervariable CDR3β loop, which suggests a potential role of this loop in fine-tuning the interactions with the F′-pocket of CD1b. Similarly the Glu68Ala mutant moderately impacted the GEM42 TCR affinity, while being critical for the GEM21 TCR recognition. More generally, the patterns of mutation that affected binding were shared between the two TCRs, pointing to a common energetic hot spot on CD1b, which coincided with the observed GEM42 TCR α-chain footprint.

### Analysis of polyclonal T cells from subjects with latent tuberculosis

**Figure 6:**
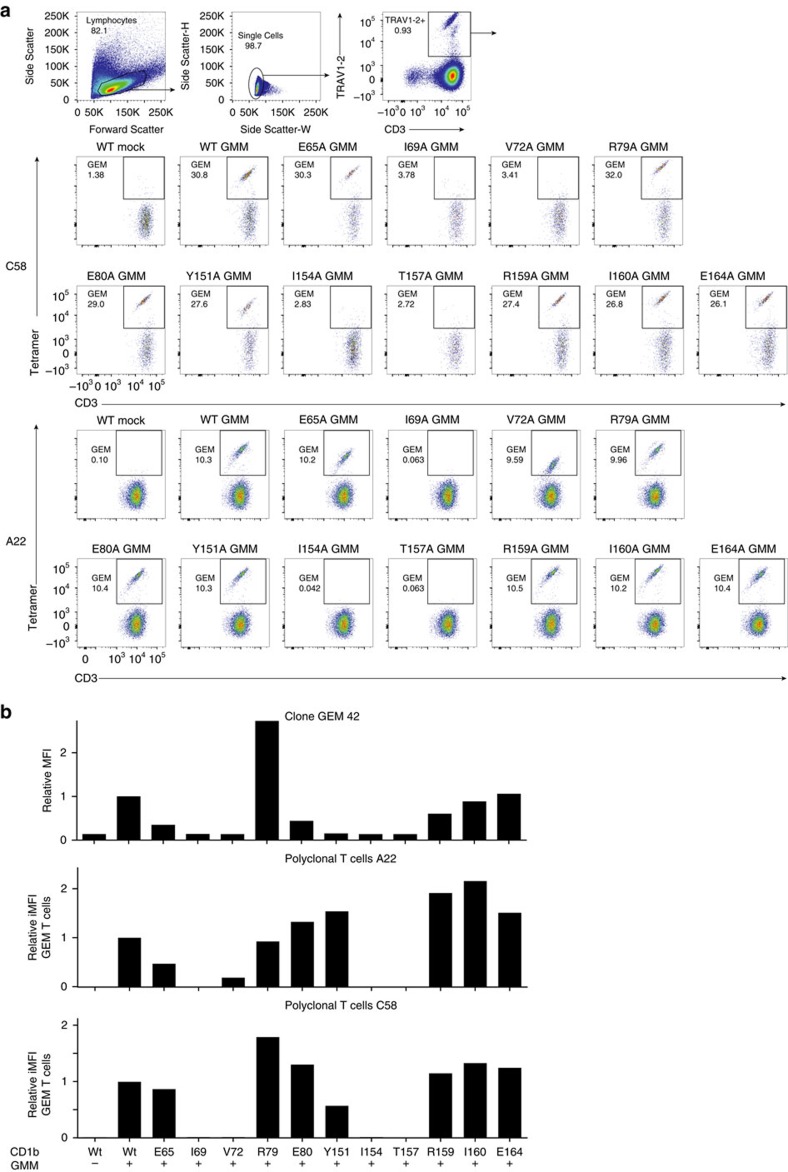
CD1b–GMM binding of Polyclonal GEM T-cells from TB patients. (**a**) Mean fluorescence intensity (MFI) of GEM clone 42, stained with untreated and GMM-loaded wild type CD1b tetramers and with eleven GMM-treated CD1b proteins, where the indicated residue was mutated to alanine. T cells from latent tuberculosis donors C58 and A22 were stimulated for one round with GMM and stained. CD3^+^TRAV1-2^+^ stimulated T cells were then used against our panel of CD1b mutants tetramer loaded with GMM. FACS plot of wild-type CD1b without GMM (WT mock) and with GMM (WT GMM), as well as for eleven CD1b mutants loaded with GMM is represented for both donors. (**b**) MFI for the GEM clone 42 or for polyclonal T cells from two donors is represented for either wild type CD1b (WT) with (+) or without (−) the presence of GMM, as well as for each CD1b mutant with (+) GMM as per the *x*-axis description. MFI and percentage GEM T cells were determined and multiplied to obtain the integrated MFI (iMFI) from the both donors. Relative MFI or relative iMFI was calculated by dividing the value obtained with a mutant tetramer by the value obtained with the wild type CD1b tetramer.

Next, we loaded 11 mutant CD1b proteins with GMM and generated a panel of fluorescent CD1b–GMM mutant tetramers. As expected, treating GEM clone 42 with wild type CD1b tetramers showed bright staining that was dependent on loading CD1b with GMM (Fig. 6). The pattern of the position of CD1b mutation could be discerned (Supplementary Table 4), with loss of staining by CD1b mutants Ile154Ala, Val72Ala, Thr157Ala and Ile69Ala (Fig. 6). The pattern generally matched that seen in SPR assays, thereby creating a new tool to dissect the CD1b specificity of polyclonal T cells.

Polyclonal GEM T cells were obtained from two unrelated *Mycobacterium tuberculosis*-infected subjects, A22 and C58, which had been expanded with autologous CD1b^+^ APCs and GMM for one round (Fig. 6). Co-staining with anti-CD3, anti-TRAV1-2 and CD1b–GMM tetramers identified triple positive GEM T cells, which comprised between 10 and 31% of all TRAV1-2^+^ cells. Strikingly, for every mutant in both patients, any effects of mutation seemed homogenous among each T cell population, rather than generating a smear from high to low staining. Thus, despite the sequence differences observed among GEM TCRs *in vivo*^20^, every CD1b mutant affects nearly all GEM T cells in a similar way, suggesting homogeneity in their TCR binding to CD1b (Fig. 6). Second, with the possible exception of Val72Ala, the position of the mutation on CD1b affected staining in both subjects in a similar way. Third, the observed pattern for polyclonal T cells largely matched (Fig. 6), those seen previously for GEM42 and GEM21 TCRs (Fig. 5) and also matched the pattern of residues making TCR contacts in the ternary structure (Fig. 2; Supplementary Fig. 7). We therefore conclude that polyclonal GEM TCRs that recognize CD1b–GMM use relatively conserved molecular mechanisms, and the GEM42 TCR is representative of *in vivo* GEM T cell populations.

## Discussion

The antigen display platform present in all four human CD1 proteins is asymmetric. When depicted in the conventional way, the ‘left' side of CD1 platforms are dominated by the A′-roofs, while the ‘right' side of CD1 platforms is characterized by a small, round portal through which antigens can protrude^4^,^12^,^13^,^14^,^33^. This feature suggests a hypothetical mechanism whereby TCRs approaching the ‘left' side would mainly contact CD1 itself, but a central or ‘right'-sided footprint would overlie the F′-portal and contact protruding antigen^4^. Indeed, a CD1a-autoreactive TCR bound directly to the A′-roof of CD1a such that its left-shifted footprint made no contact with a lipid ligand^23^. In contrast the GEM42 TCR adopts a central position and extensively contacts the glycolipid, thereby revealing that highly distinct TCR recognition mechanisms exist in the group 1 CD1 system.

GEM T cells are defined by the particular TRAV1-2^+^ TCR they express^20^. These data using tetramers with mutant CD1b proteins demonstrate that the pattern of CD1b-lipid complex recognition is conserved among polyclonal T cells from unrelated donors. The GEM TCRs specificity towards GMM is notable given that GMM arises in mycobacteria from the esterification of host glucose with mycobacterial-derived mycolic acid, and thus the existence of GMM is considered to represent a signature for mycobacterial infection of hosts^34^. Indeed, the intricate network of interactions provide a basis for understanding the strict specificity of the GEM42 TCR towards GMM, including its C2 and C4 hydroxyl groups of the glucosyl moiety. GMM is a foreign lipid that is structurally distinct from all known self lipids. Although self mono-glucosyl lipids exist, the 6-linked glucose present in GMM is pivoted in a way that differs substantially from 1-linked glucose in β-glucosyl ceramides. For Type I and Type II NKT TCR recognition of glycolipids, the TCR α and TCR-β chains, respectively, dominate contacts with the glycolipid headgroup^25^,^26^. Here the TCR α- and β-chains function together so that the headgroup lies between the two chains, acting like tweezers. Thus GEM TCRs are exquisitely and simultaneously sensitive to two fixed components, namely CD1b and the mycobacterial glycolipid antigen itself. In contrast to broad expression of antigens for MAIT and NKT cells, GEM T cells are specific for an antigen found only in a limited range of Actinobacteria and so detection or manipulation of their response could serve diagnostic or therapeutic purposes.

The GEM TCR–CD1b–GMM structure provided specific insights into both the function of aspects of the TCR that are highly conserved (TRAV1-2, TRAJ9) and less conserved (TRBV6-2). Moreover, CD1b mutagenesis studies showed that the ‘energetic hot spot' matched with the invariant TCR α-chain footprint of two characterized GEM TCRs, as well as polyclonal GEM T cells isolated from tuberculosis patients. Thus GEM T cell recognition is underpinned by a conserved CD1b–GMM footprint driven by the invariant TCR α-chain usage. As CD1b can present mycolates of different length (C32-C80), the impact of such variation could effect GEM TCR recognition by altering the conformation of CD1b platform itself. We observed a conserved footprint of TRAV1-2 encoded residues in GEM T cells, MAIT cells and MHC-reactive T cells, which was surprising based on the markedly differing primary structures of these three antigen-presenting molecules. The conserved mode of binding is not determined by individual, residue-specific interactions. Instead, key contacts are made with a shared aspect of tertiary structure, highlighting the versatility of this scaffold in recognizing distinct classes of antigen-presenting molecules. In summary, here we report the first structure of an αβ TCR bound to CD1b–antigen complex, and simultaneously provide a molecular basis into how the immune system uses conserved recognition features to target a mycobacterial lipid antigen formed during infection of the host.

## Methods

### Lipid antigens

Lipids from *Rhodococcus equi* and *Mycobacterium phlei* (American Type Culture Collection) were isolated using prior methods^28^,^34^ with minor modifications, including supplementation of with 30 g per litre d-glucose, D-(+)-mannose, D-(+)-galactose, or glycerol to influence the glycolipids produced. The bacteria were harvested, washed by PBS twice and distilled water once, followed by chloroform (CHCl_3_)/methanol (MeOH) extraction. After loading onto an open silica column, lipids were eluted with an CHCl_3_/acetone gradient. C32 GMMs were found in 50–60% acetone in CHCl_3_ fractions. C80 GMMs, C80 mannose monomycolates, and C80 galactose monomycolates were found in 30% acetone fractions. C80 glycerol monomycolates were found in 10–15% acetone in CHCl_3_ column fractions. The column fractions with the desired glycolipid were further purified by one dimensional thin layer chromatography using silica-coated glass plates (Scientific Absorbents). After plates were precleared in CHCl_3_/MeOH/H_2_O 60:30:6 (v/v/v), hexosyl mycolates were purified by preparative TLC with CHCl_3_/MeOH/H_2_O 60/16/2 (v/v/v). Glycerol monomycolates were purified with CHCl_3_/MeOH 97/3. Glycolipids were validated by nanospray ESI mass spectrometry performed on the LXQ ion-trap mass spectrometer. C80 mycolic acids (#M4537) were from Sigma-Aldrich, Natick, MA.

### Protein expression, purification and crystallization

The GEM42 and GEM21 TCRs were expressed as inclusion body, and then refolded in a cold buffer containing 5 M urea, 400 mM L-Arginine-HCl, 100 mM Tris–HCl pH 8, 2 mM EDTA pH 8, 0.5 mM oxidized glutathione and 5 mM reduced glutathione. The TCRs were then dialyzed three times against 10 mM Tris–HCl pH 8 over 24 h, and purified by anion exchange and size exclusion chromatography^20^. CD1b was expressed either in High Five insect cell lines or HEK 293S GnTI^-^ cells (from ATCC). Alanine mutants of the CD1b molecules were generated by site directed mutagenesis and expressed in mammalian cells as per the wt CD1b molecule. The CD1b molecule and mutants were firstly loaded using C16 lyso-sulfatide lipid (from Matreya, LLC). The charged wt or mutant CD1b-lyso-sulfatide was then purified over an anion exchange column. Subsequently the CD1b-lyso-sulfatide was loaded with the C32 GMM lipid and the CD1b–GMM complex (and mutants thereof) was purified over an anion exchange column.

### Crystallization and structure determination

Crystals of the CD1b–GMM complex were grown by the hanging-drop, vapour-diffusion method at 20 °C with a protein/reservoir drop ratio of 1:1, at a concentration of 10 mg ml^−1^ in 10 mM Tris–HCl pH 8, 150 mM NaCl using 20–30% PEG 4 K, 0.2 M Na Iodide and 2% ethylene glycol. Crystals of the GEM42 TCR in complex with the CD1b-Ile160Ala-GMM were obtained at a concentration of 2 mg ml^−1^ using 1.5–2 M NH_4_SO_4_, 0.1 M Tris–HCl pH8.5, 10 mM MgCl_2_ and 10 mM CeCl_2_.

Crystals were soaked in a cryoprotectant solution containing mother liquor solution with PEG4000 concentration increased to 30% (v/v) for the CD1b–GMM crystals and 20% ethylene glycol for the ternary complex crystals, and flash frozen in liquid nitrogen. Data were collected (at 100 K) on the MX2 beamline at the Australian Synchrotron, using the ADSC-Quantum 315r CCD detectors (at 100 K). Data were processed with XDS software^35^, and scaled using SCALA software from the CCP4 suite^36^. Due to the weak diffraction of the ternary complex crystals, multiple crystals (∼10–15) were merged together, resulting in a multiplicity of almost 70. This allowed a gain in intensity, resolution as well as having better 2Fo-Fc and mFo-Fc density maps for the lipid antigen (Table 1). In a similar fashion, the CD1b–GMM data set represents a 360° sweep of data to improve the density of the lipid by increasing the multiplicity from a single crystal. The density for the acyl tails and the ester were clear; however the glucose moiety is solvent exposed and highly mobile. The glucose is most likely adopting a multitude of alternate conformations. Due to the high resolution and high multiplicity, we were able to build one conformation described here (Supplementary Fig. 4) that represents ∼20–30% occupation of the glucose moiety and was the only clear conformation that we could build. In turn the glucose is bulging out of the cleft, and is not stabilised by interaction with either the CD1b molecule itself or crystal packing, resulting in a mobile head group and weak density around it. The GEM42 TCR structure was determined by molecular replacement using PHASER program with the previously solved free GEM42 TCR as the search model for the TCR (Protein Data Bank accession number, 4G8F (ref. 20)), and we used previously solved CD1b structure as the search model (Protein Data Bank accession number, 1UQS (ref. 29)). Manual model building was conducted using the Coot software^37^ followed by maximum-likelihood refinement with Buster^38^. The final models have been deposited to the Protein Data Bank under the accession code: 5L2J for CD1b–GMM (Ramachandran plot: 0% outlier) and 5L2K for GEM42 TCR–CD1b–GMM (Ramachandran plot: 0.9% outlier), and the final refinement statistics are summarized in Table 1. All molecular graphics representations were created using PyMol (DeLano WL. The PyMOL Molecular Graphics System. http://www.pymol.org 2002)^39^. The buried surface area is calculated with the AreaIMol program, the contacts generated by the Contact program, both form the CCP4 suite. The structures used to generate the comparison and figures are the MAIT-MR1-metabolite (PDB code: 4PJ7) (ref. 40); ELS4-MHC-peptide (PDB code: 2NX5) (ref. 30); BK6-CD1a-lipid (PDB code: 4X6C) (ref. 23) and RL42-MHC-peptide (PDB code: 3SJV) (ref. 41).

### Surface plasmon resonance measurement and analysis

Surface plasmon resonance experiments were conducted at 25 °C on the BIAcore 3,000 instrument with TBS buffer (10 mM Tris–HCl, pH 8, 150 mM NaCl) and was supplemented with 1% BSA^42^. The GEM42 and GEM21 TCRs (maximum concentration of 20 μM) were used as analytes, while the CD1b–lipid complexes were amine coupled to CM5 chips. BIAevaluation Version 3.1 was used for data analysis using the 1:1 Langmuir binding model. Experiments were carried out in duplicate (*n*≥2), with the wild type CD1b–GMM complexes as positive controls and CD1b-endogenous lipid as negative controls.

### T cell assays

Peripheral blood mononuclear cells (PBMCs) from tuberculin positive subjects with no evidence of active tuberculosis were obtained after informed consent, with oversight of institutional review boards of the Lemuel Shattuck Hospital and Partners Healthcare. PBMC (50 × 10^6^) from two latent tuberculosis patients (A22 and C58) were stimulated with 20 × 10^6^ autologous GM-CSF and IL-4 treated monocytes and 2 μg ml^−1^ *M. phlei* GMM and cultured for 20 days in the presence of 1 nM human IL-2 (BD Pharmingen, catalogue number 554424 and 554426). The resulting cells were stained with Allophycocyanin-labeled, GMM-loaded CD1b tetramers for 10 min at room temperature, followed by addition of anti-CD3-Fluorescein (clone SK7, Becton Dickinson, catalogue number 349201, was used at a dilution of 1:12.5) for 10 min at room temperature, followed by addition of anti TRAV1-2-Phycoerythrin (clone 3C10 from Biolegend, catalogue number 351706, was used at a dilution of 1:62.5) for 20 min on ice, followed by a wash and analysis on a 5-laser BD Fortessa flow cytometer. After gating for live, single lymphocytes based on forward and side scatter properties, CD3^+^tetramer^+^TRAV1-2^+^ were isolated, and integrated mean fluorescence (iMFI) was calculated by multiplying the percentage of T cells in the lymphocyte gate with the tetramer MFI. The variable regions of the human TCRα- and TCRβ-chains from GEM clones 18 and 42 were cloned in-frame with the respective constant regions of mouse TCRs into retroviral vectors. The respective TCR sequences of the α and β chains from clone 42 and 18 were cloned into mouse stem cell virus-based retroviral plasmids with an internal ribosomal entry site plus sequences encoding either green fluorescent protein or human nerve growth factor receptor as reporters. All TCR constructs were expressed by retroviral transduction in 5KC-78.3.20, a hybridoma clone selected for loss of both TCRα and TCRβ. TCR constructs in retroviral plasmids were transfected into Phoenix cells together with the pCLEco accessory plasmid with Lipofectamine 2,000 (Invitrogen) according to the manufacturer's instructions. Retrovirus-containing supernatants were collected 48 h after transfection and were centrifuged to remove cell debris. Hybridomas (1 × 10^5^ cells) were ‘spin-infected' at 3,300 *g* for 90 min at 37 C in 1.5-ml microcentrifuge tubes along with retroviral supernatants containing polybrene (8 mg ml^−1^). After ‘spin infection', hybridomas were sorted on a MoFlo cell sorter (Dakocytomation) on the basis of retroviral reporter and TCRβ expression^43^. The capacity of GMM analogues to stimulate clone 18 and 42T cells was determined by incubating 10^5^ hybridoma cells with 5 × 10^4^ allogeneic GM-CSF and IL-4 treated monocytes in the presence of 10 μg ml^−1^ of lipid and 20 ng ml^−1^ phorbol 12-myristate 13-acetate. After 24 h, the IL-2 concentration in the supernatant was determined by ELISA.

### Data availability

The final model has been validated using the Protein Data Base validation web site and the coordinates relating to the data reported in this study are deposited in the PDB database, code: 5L2J for CD1b–GMM structure and 5L2K for GEM42 TCR–CD1b–GMM complex structure. All remaining data are available within the article and its supplementary information files and from the corresponding authors on reasonable request.

## Additional information

**How to cite this article:** Gras, S. *et al*. T cell receptor recognition of CD1b presenting a mycobacterial glycolipid. *Nat. Commun.* **7,** 13257 doi: 10.1038/ncomms13257 (2016).

**Publisher's note:** Springer Nature remains neutral with regard to jurisdictional claims in published maps and institutional affiliations.

## Supplementary Material

Supplementary Information Supplementary Figures 1-7, Supplementary Tables 1-4, Supplementary References.

## Figures and Tables

**Table 1:** Data collection and refinement statistics.

	**CD1b**–**GMM**	**GEM 42 TCR**–**CD1b**–**GMM**
*Data collection*		
Space group	*P*2_1_2_1_2_1_	*P*6_4_22
Cell dimensions		
*a*, *b*, *c* (Å)	57.66, 78.11, 91.33	175.00, 175.00, 170.87
Resolution (Å)	57.66–1.65 (1.68–1.65)	48.45–3.20 (3.37–3.20)
*R*_pim_* (%)	4.5 (67.0)	11.4 (99.2)
CC_1/2_ (%)	99.8 (58.4)	99.6 (63.8)
I/σ_I_	13.6 (2.1)	14.4 (1.8)
Data completeness (%)	100.0 (100.0)	100.0 (100.0)
Multiplicity	13.0 (13.2)	67.7 (61.1)
*Refinement*		
Resolution (Å)	35.91–1.65	43.75–3.20
No. reflections	50306	26029
*R*_*factor*_†^/^*R*_*free*_† (%)	17.3/19.8	18.5/23.1
No. atoms		
Protein	3072	6383
Ligand/ion	180	100
Water	367	6
B-factors		
Protein	23.1	84.3
Ligand/ion	37.3	106.0
Water	34.0	45.3
Rms deviations from ideality		
Bond lengths (Å)	0.010	0.010
Bond angles (°)	1.06	1.27
Ramachandran plot (%)		
Allowed region	100.0	99.1
Disallowed region	0	0.9

^*^R_p.i.m_=Σ_hkl_ [1/(*N*−1)]^1/2^ Σ_i_ | *I*_hkl, i_ −<*I*_hkl_>|/Σ_hkl_ <*I*_hkl_>.

^†^*R*_factor_=Σ_hkl_||*F*_o_|−|*F*_c_||/Σ_hkl_|*F*_o_| for all data except≈5% which were used for *R*_free_ calculation. Highest resolution shell is shown in parenthesis.
