# Supplementary material for: T cell receptor recognition of CD1b presenting a mycobacterial glycolipid
Source: Nat Commun. 2016 Oct 27;7:13257. doi: 10.1038/ncomms13257 (PMC5095289; doi:10.1038/ncomms13257)
Supplement: Supplementary Information — Supplementary Figures 1-7, Supplementary Tables 1-4, Supplementary References. [file ncomms13257-s1.pdf]

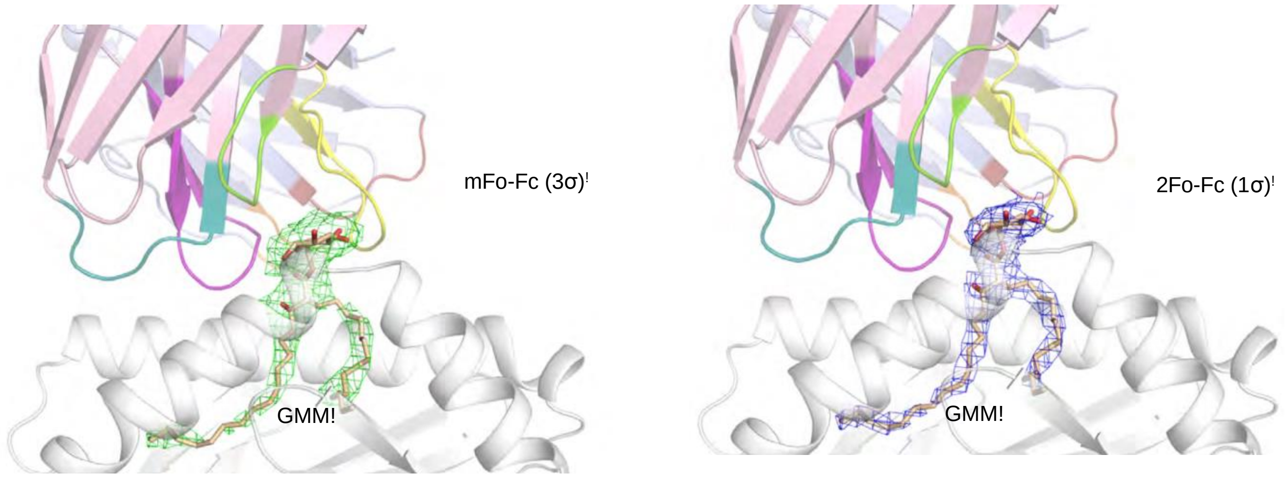


**Supplementary Figure 1.**

Electron density map of the GMM-lipid complex at the interface of the CD1b and GEM42 TCR. The GEM42 TCR is coloured in pale pink for the α-chain and pale blue for the β-chain, with the CDR1, CDR2 and CDR3 loops coloured in blue, green and yellow, respectively, for the α-chain and red, orange and yellow for the β-chain. The CD1b is coloured in white and the GMM is represented in pale orange stick. The left panel represents the unbiased omit map (Fo-Fc) at 3σ in green mesh, while the right panel shows the refined map (2Fo-Fc) at 1 σ in blue mesh.

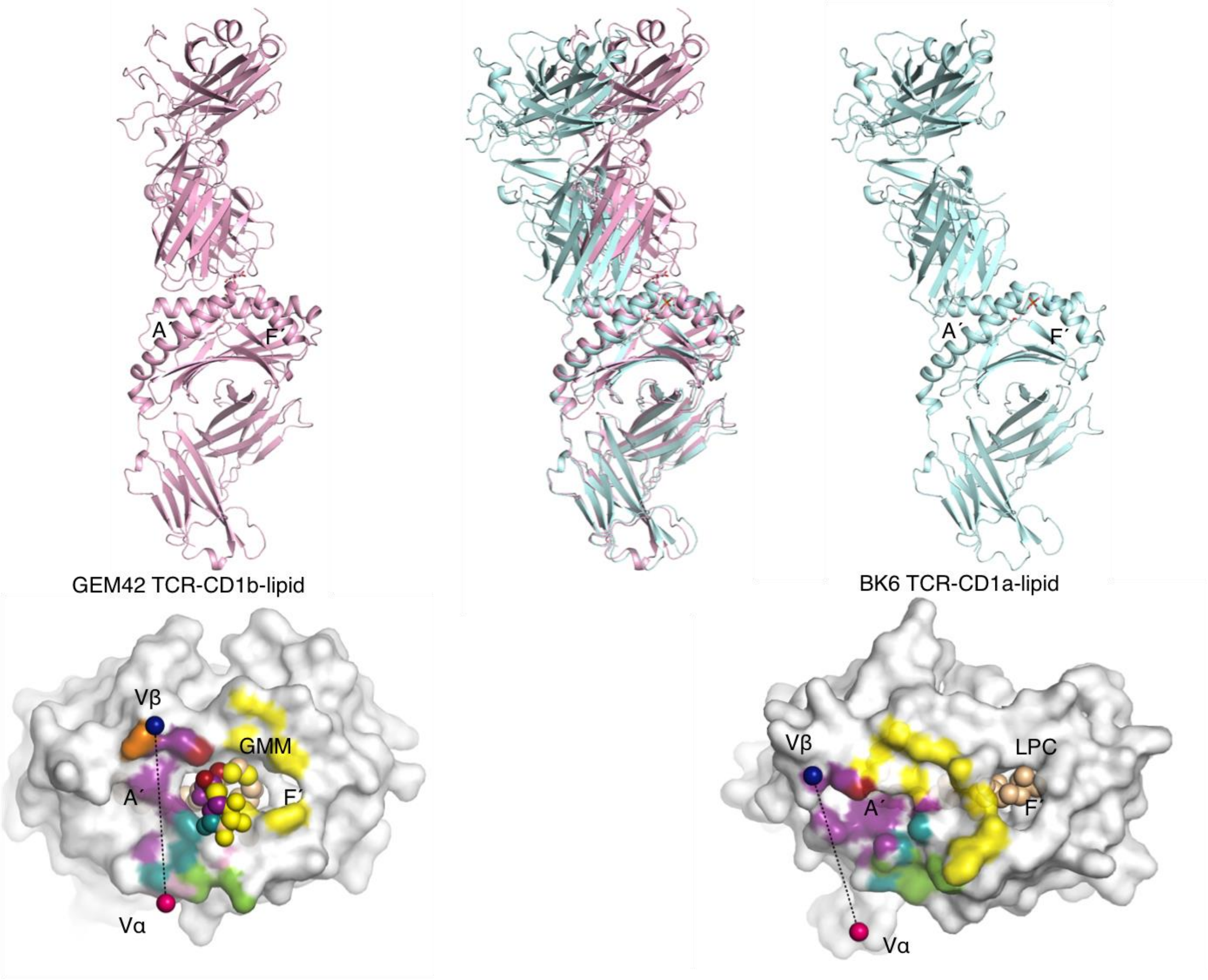


**Supplementary Figure 2.**

Comparison of the GEM42 TCR-CD1b-GMM complex structure in pink (left side) with the BK6 TCR-CD1a-LPC complex in pale blue (right side), with an overlay of both complexes. The bottom panels represent the footprint of the respective TCR, coloured by atomic contacts with the TCR, namely α-chain and pale blue for the β-chain, with 3 CDR loops coloured in blue, green and yellow for the α-chain and red, orange and yellow for the β-chain, respectively. The pink and blue spheres represent the mass centre of the Vα and Vβ domains, while the pale orange sphere represent the lipid antigen.

```
     !     !     !     !  70          80          90          100         110!
                          |           |           |           |           |           !
CD1b       DKEVAELEEIFRVYIFGFAREVQDFAGDFQMKYPFEIQGIAGCELHSGGAIVSFLRGALG!
CD1c       NEELSDLELLFRFYLFGLTREIQDHASQDYSKYPFEVQVKAGCELHSGKSPEGFFQVAFN!
CD1a       NEEWKELETLFRIRTIRSFEGIRRYAHELQFEYPFEIQVTGGCELHSGKVSGSFLQLAYQ!
CD1d       DQQWETLQHIFRVYRSSFTRDVKEFAKMLRLSYPLELQVSAGCEVHPGNASNNFFHVAFQ!
 !
     !     !120 !     !  130!     !  140!     !  150!      160      !  170      180
     !     !  |           |           |            |          |       !   |  !      |!
CD1b       GLDFLSVKNASCVPSPEGGSRAQKFCALI-IQYQGIMETVRILLYETCPRYLLGVLNAGK!
CD1c       GLDLLSFQNTTWVPSPGCGSLAQSVCHLLNHQYEGVTETVYNLIRSTCPRFLLGLLDAGK!
CD1a       GSDFVSFQNNSWLPYPVAGNMAKHFCKVL-NQNQHENDITHNLLSDTCPRFILGLLDAGK!
CD1d       GKDILSFQGTSWEPTQEAPLWVNLAIQVL-NQDKWTRETVQWLLNGTCPQFVSGLLESGK!
    !
```

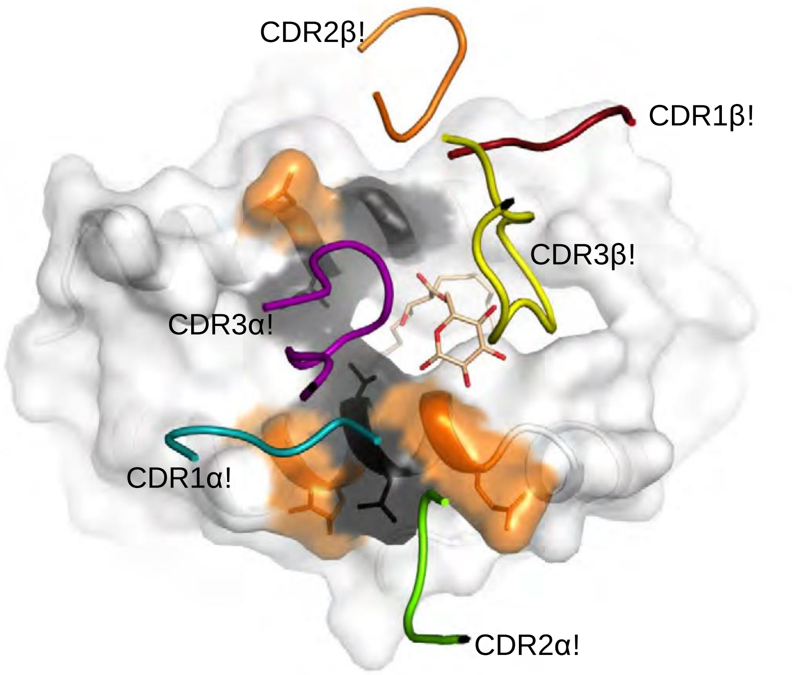


**Supplementary Figure 3.**

Sequence alignment of human CD1a, CD1b, CD1c and CD1d heavy chains. The red residues indicate the CD1b amino acid forming interaction with GEM42 TCR. Underlined and red indicates interaction with the α-chain of GEM42 TCR. The orange residues indicate the residues from CD1b involved in the interaction with the GEM42 TCR and not conserved with some of the other CD1 heavy chains. The bottom panel show a surface representation of the CD1b-GMM in complex with the GEM42 TCR (represented by CDR loops). The CD1b residues involved only with the GEM42 TCR α-chain are coloured either in orange (if not conserved with other CD1 molecules) or black (if conserved with other CD1 molecules). The GEM42 TCR CDR loops are coloured teal, green and purple for the CDR1, 2 and 3α; and red, orange and yellow for the β-chain.

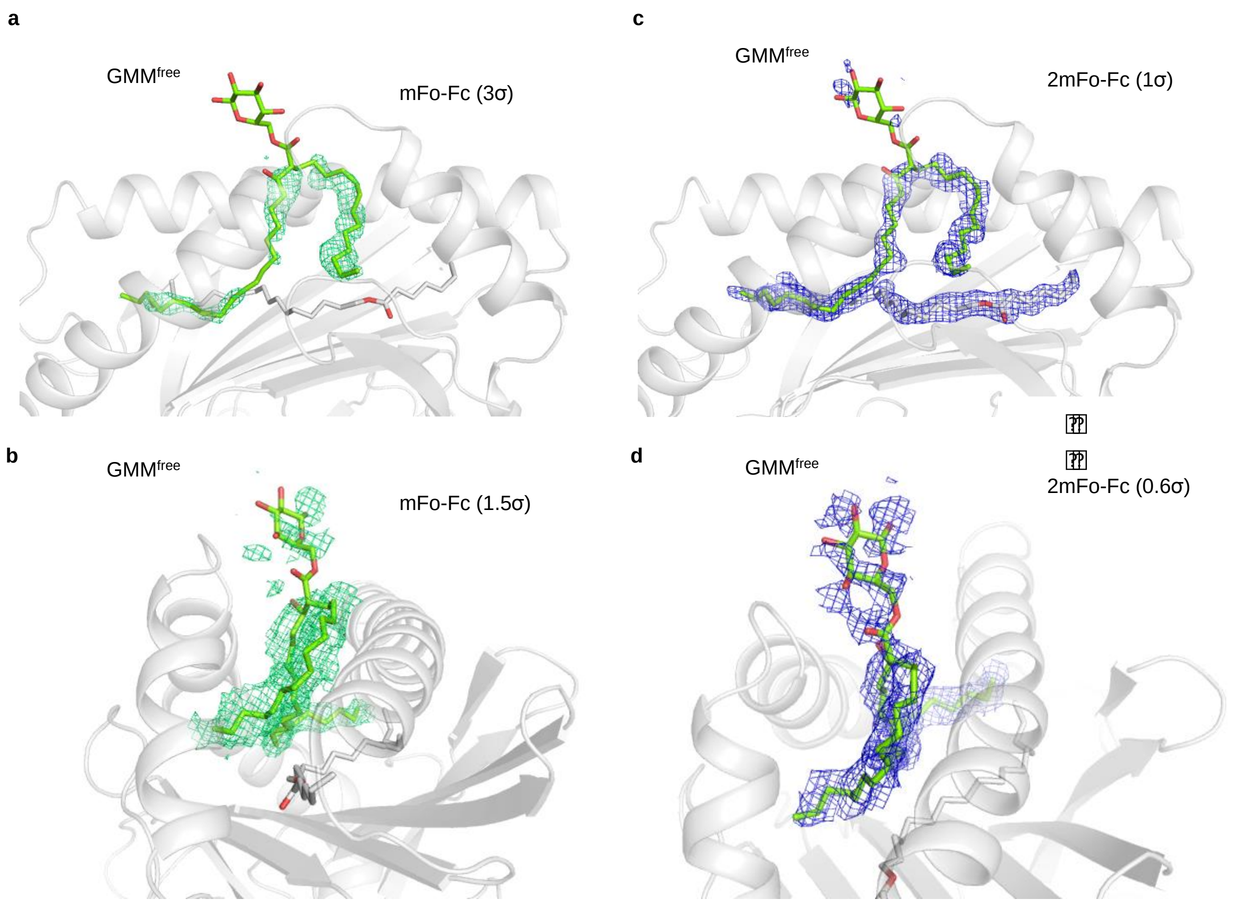


**Supplementary Figure 4.**

Within the CD1b-GMM binary structure, while the electron density for the acyl chains were clear, the electron density corresponding to glucose headgroup was poor, indicating mobility of this moiety. Representation of the CD1b in white cartoon and GMM in green stick from the binary CD1b-GMM-C32 structure. The electron density map is represented in green mesh for the unbiased omit map Fo-Fc at 3σ (a) and 1.5σ (b), while the 2Fo-Fc map are coloured in blue mesh and represented at 1σ (c) and 0.6σ (d).

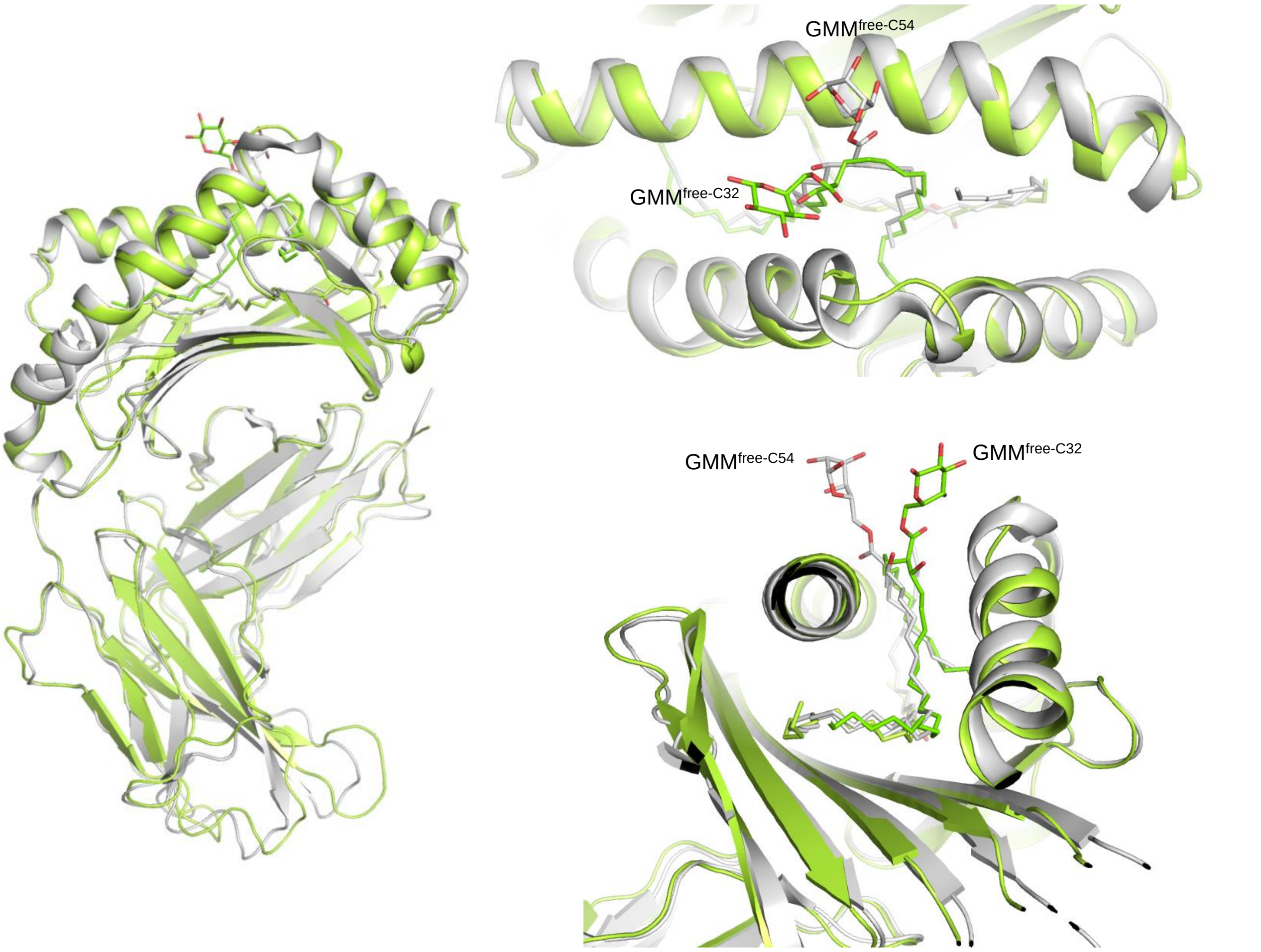


**Supplementary Figure 5.**

Superimposition of the free structure of CD1b represented in cartoon bound to either the C32 GMM (green) or C54 GMM (white), with in both cases the GMM represented in stick. The different panels represent different view of the same superimposition.

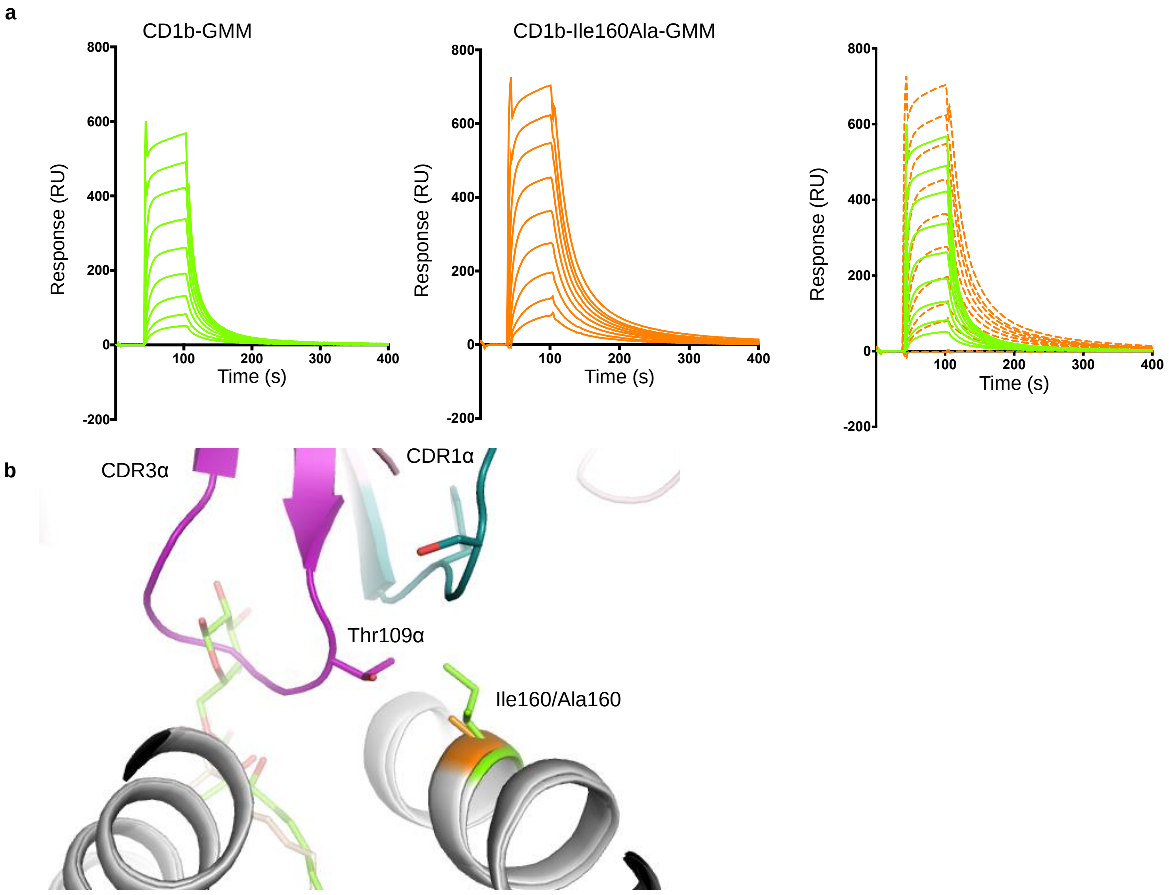


**Supplementary Figure 6.**

(a) Surface plasmon resonance (SPR) sensogram of a range of concentration of GEM42 TCR onto the CD1b-GMM (green curves) or CD1b-Ile160Ala-GMM (orange curves) extracted from the same experiment. The right panel show an overlay of the curves showing the slower off rate of the GEM42 TCR when binding to the CD1b-Ile160Ala compare to the CD1b- GMM complex.

(b) Superimposition of the binary CD1b-GMM structure and in complex with the GEM42 TCR, the CD1b is represented in white cartoon, the Ile160 from the free CD1b-GMM structure is in green stick and the Ala160 form the GEM42 TCR-CD1b-GMM complex is in orange stick. The CDR3α is in purple, the CDR1α in teal, and the Thr109α is represented as purple stick.

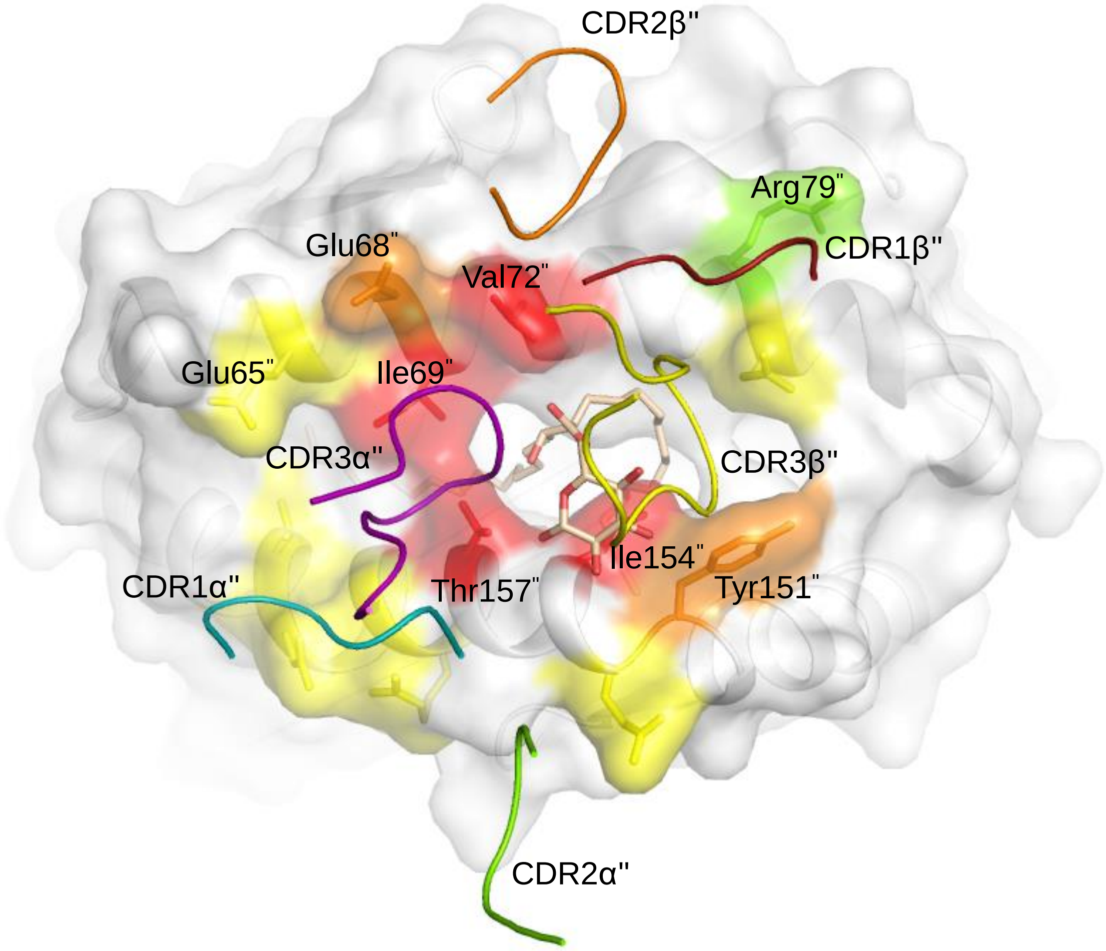


**Supplementary Figure 7.**

Surface representation of CD1b (white) and GMM (pale orange stick) with the mutated residues coloured as per their impact on the GEM42 TCR affinity, ranging from 3-fold improvement (green), no impact (yellow), 3-fold decrease (orange) and more than 5-fold decrease (red). The CDR loops of the GEM42 TCR are represented as loop and coloured in teal, green and purple for the α-chain and red, orange and yellow for the β-chain.

**Supplementary Table 1. GEM TCR sequences.**

| GEM TCR | TRAV | CDR1α | CDR2α | CDR3α TRAV1-2 TRAJ9 |
|---|---|---|---|---|
| **GEM42** | 1-2*01 | TSGFNG | NVLDGL | A V R **N** T G G F K T I<br>gct gtt cga aat act gga ggc ttc aaa act atc |
| **GEM18** | 1-2*01 | TSGFNG | NVLDGL | A V L **N** T G G F K T I<br>gct gtc cta aat act gga ggc ttc aaa act atc |
| **GEM21** | 1-2*01 | TSGFNG | NVLDGL | A V R **R** T G G F K T I<br>gct gtg aga agg act gga ggc ttc aaa act atc |
| **GEM1** | 1-2*01 | TSGFNG | NVLDGL | A V R **V** T G G F K T I<br>gct gtg aga gtc act gga ggc ttc aaa act atc |
| **A14 PCR1** | 1-2*01 | TSGFNG | NVLDGL | A V R **G** T G G F K T I<br>gct gtg agg ggt act gga ggc ttc aaa act atc |
| **A14 PCR2** | 1-2*01 | TSGFNG | NVLDGL | A V R **N** T G G F K T I<br>gct gtg aga aat act gga ggc ttc aaa act atc |

| GEM TCR | TRBV | CDR1β | CDR2β | CDR3β |
|---|---|---|---|---|
| **GEM42** | 6-2*01 | MNHEY | SVGEGT | ASSPRLAG**D**EQFF |
| **GEM18** | 6-2*01 | MNHEY | SVGEGT | ASRPPLTARGLKHTGELFF |
| **GEM21** | 30*01 | GTSNPN | SVGIG | AWAKTGFGG**D**TQYF |
| **GEM1** | 30*01 | GTSNPN | SVGIG | AWSPTSLRLAST**D**TQYF |

All sequences have been reported previously[1] . Two sequences were derived from PCR on single CD1b-GMM tetramer+ T cells (A14 PCR1 and 14 PCR2), and for these, no TCRβ chain is known. The residue coloured in red is the Arg107α characteristic of the GMM-specific GEM T cells, the bold residues represent the variable residue at position 108α. The Asp113β of GEM42 TCR (blue and bold) is conserved with GEM21 and GEM1 TCRs.

**Supplementary Table 2. Contacts between GEM42 TCR and CD1b-GMM**

| TCR gene | PDB | CD1b | Bond type |
|---|---|---|---|
| CDR1α | Phe30-O | Arg159-NH2 | HB |
| CDR1α | Asn31-Oδ1 | Gly153-O, Glu156, Thr157-N-Oγ1 | HB, VDW |
| FWα | Tyr55-OH | Gly153-N | VDW, HB |
| CDR2α | Val57 | Gln152, Glu156 | VDW |
| CDR2α | Leu58 | Gln152, Glu156 | VDW |
| FWα | Arg84-NH1-NH2 | Glu156-Oε1, Oε2, Arg159 | SB, VDW |
| CDR3α | Thr109 | Ile69, Ala160 | VDW |
| CDR3α | Gly110 | Thr157 | VDW |
| CDR3α | Phe112 | Glu68, Ile69, Val72 | VDW |
| CDR1β | Tyr31 | Val72 | VDW |
| FWβ | Tyr55-OH | Glu68-Oε1-Oε2 | SB, VDW |
| CDR2β | Val57 | Val72 | VDW |
| FWβ | Ala67 | Glu68 | VDW |
| CDR3β | Arg109-Nε-NH2 | Phe75, Gly76, Arg79, Glu80-Oε1 | SB, VDW |
| CDR3β | Leu110 | Glu80, Tyr151, Ile154 | VDW |
| CDR3β | Ala111 | Tyr151 | VDW |
| **TCR gene** | **PDB** | **GMM** | **Bond type** |
| CDR1α | Asn31-Nδ2 | 1-OH | HB, VDW |
| FWα | Tyr55 | 2-OH | VDW |
| CDR3α | Arg107-NH2 | C1, 2-OH | HB, VDW |
| CDR3α | Gly110-O | C7, 7-O, C9, 9-OH | HB, VDW |
| CDR3α | Gly111 | C1, 5-O, C5, C6 | VDW |
| CDR3α | Phe112 | 7-O, | VDW |
| CDR1β | Tyr31-OH | C7, CAQ, 7-O1, C6 | HB, VDW |
| CDR3β | Pro108 | 4-OH | VDW |
| CDR3β | Arg109-O | CAR, C4, 4-OH, C6, 6-O | HB, VDW |
| CDR3β | Leu110 | CAT, | VDW |
| CDR3β | Ala111-N | C4, 4-OH, C3, 3-OH | HB, VDW |
| CDR3β | Gly112-N | 4-OH, | HB, VDW |
| CDR3β | Asp113-Oδ2-Oδ1 | 2-OH, C3, 3-OH | VDW, HB |

VDW: Van der Waals, HB: Hydrogen bond, SB: salt bridge.

**Supplementary Table 3.**

| TRAV1-2 | CD1b-lipid | MHC-peptide | MR1-metabolite |
|---|---|---|---|
| **Ser28 (CDR1α)** | - | Glu166 | - |
| **Gly29 (CDR1α)** | - | Leu163, Glu166 | Glu160 |
| **Phe30 (CDR1α)** | Arg159 | - | Glu160, Asn155 |
| **Asn31 (CDR1α)** | Gly153, Glu156, Thr157, lipid | Ala158, Leu163, peptide | - |
| **Tyr55 (FWα)** | Gly153, lipid | Glu154 | His148, Tyr152 |
| **Val57 (CDR2α)** | Gln152, Glu156 | Ala158 | Leu151,TyrY152, Asn155 |
| **Leu58 (CDR2α)** | Gln152, Glu156 | Arg157 | Leu151, Lys154, Asn155 |
| **Arg84 (FWα)** | Glu156, Arg159 | Gly162 | Asn155, Glu159 |

The table summarises the contacts made by the germline encoded TRAV1-2 residues with CD1b-lipid (white), MHC-peptide (dark gray) and MR1 (gray), coloured accordingly to the bottom panels of Figure 1. FW indicate framework residues.

**Supplemental Table 4. SPR with CD1b mutants**

| CD1b-GMM | GEM 42 Kd (μM) | ΔΔGeq (kcal.mol$^{-1}$) | GEM 21 Kd (μM) | ΔΔGeq (kcal.mol$^{-1}$) |
|---|---|---|---|---|
| CD1b-WT | **1.15 ± 0.01** | | **0.70 ± 0.02** | |
| **CD1b mutant** | | | | |
| E65A | **1.92 ± 0.02** | 0.300 ± 0.003 | **2.14 ± 0.06** | 0.669 ± 0.008 |
| E68A | **5.46 ± 0.16** | 0.922 ± 0.020 | **12.21 ± 0.05** | 1.690 ± 0.001 |
| I69A | **> 20** | > 1.690 | **6.09 ± 0.03** | 1.282 ± 0.001 |
| V72A | **13.22 ± 1.07** | 1.420 ± 0.025 | **4.60 ± 0.64** | 1.153 ± 0.038 |
| R79A | **0.35 ± 0.01** | -0.712 ± 0.008 | **1.00 ± 0.03** | 0.219 ± 0.008 |
| E80A | **1.21 ± 0.05** | 0.017 ± 0.012 | **0.92 ± 0.01** | 0.164 ± 0.003 |
| Y151A | **3.69 ± 0.09** | 0.682 ± 0.008 | **0.93 ± 0.01** | 0.171 ± 0.003 |
| Q152A | **2.57 ± 0.05** | 0.475 ± 0.011 | **1.65 ± 0.02** | 0.509 ± 0.005 |
| I154A | **NB** | >> 1.690 | **>20** | > 1.984 |
| T157A | **> 20** | > 1.690 | **> 20** | > 1.984 |
| R159A | **1.13 ± 0.01** | -0.013 ± 0.004 | **0.68 ± 0.02** | -0.008 ± 0.001 |
| I160A | **0.81 ± 0.03** | -0.218 ± 0.011 | **0.44 ± 0.01** | -0.268 ± 0.001 |
| E164A | **1.95 ± 0.01** | 0.312 ± 0.003 | **0.80 ± 0.02** | 0.078 ± 0.014 |
| T165A | **1.67 ± 0.02** | 0.220 ± 0.007 | **0.78 ± 0.02** | 0.063 ± 0.015 |

ΔΔGeq= RTln(KdeqMut/KdeqWT), WT refers to wild-type, T is the temperature of the experiment and R is the gas constant, NB refers to no binding.

## References


1. Van Rhijn I, Kasmar A, de Jong A, Gras S, Bhati M, Doorenspleet ME, *et al.* A conserved human T cell population targets mycobacterial antigens presented by CD1b. *Nat Immunol* 2013, **14**(7)**:** 706-713.
